# Ethical, moral and other aspects related to fertility preservation in cancer patients

**DOI:** 10.5935/1518-0557.20170011

**Published:** 2017

**Authors:** Bruno Ramalho de Carvalho, Jhenifer Kliemchen, Teresa K. Woodruff

**Affiliations:** 1 Bonvena - Medicina Reprodutiva, Brasília, DF, Brazil; 2 Latin America Oncofertility Network, The Oncofertility Consortium); 3 In Vitro Consultoria, Belo Horizonte, MG, Brazil; 4 Department of Obstetrics and Gynecology, Northwestern University Feinberg School of Medicine, Chicago, IL, USA

**Keywords:** Oncofertility, fertility preservation, cancer, bioethics, ethics, cryopreservation

## Abstract

Post-treatment fertility emerges as an important issue in the early counseling of individuals with cancer, since survivors may have their quality of life affected by the occurrence of functional failure of the gonads because of antineoplastic therapies. In the context, oncofertility has been developed as an interdisciplinary field of study that combines expertise in reproductive medicine and oncology, to provide strategies aiming to maintain the possibility of future procreation. Today, we have many options and techniques available for the preservation of gametes in men and women. Some of them are already considered well established and used in routine, but ethical and moral issues on the subject still need to be debated.

## CONTEXT

Having children was reported to be of interest for 76% of cancer survivors (Schover *et al*., 1999). Then, post-treatment fertility emerges as an important issue in the early counseling of individuals with cancer, since survivors may have their quality of life affected by the occurrence of functional failure of the gonads because of antineoplastic therapies. Unfortunately, to date there are no tools for predicting the occurrence or the extent of gonadotoxic damage, or even that can assess if the damage will lead to definitive inability to spontaneously conceive (Carvalho, 2015).

The negative impact of cancer treatment on reproductive physiology also has negative emotional effects. The psychological stress generated in individuals during cancer treatment can result not only from the infertility because of the treatment, but it may also be related to the symbolic loss of the idea of family completeness (Green *et al*., 2003; Carter *et al*., 2005).

The point is not to compare cancer to infertility regarding its impact on the physical health of the individuals, but both diagnoses break the ideal continuity of human beings. From the perspective of the end of the line, one can imagine the devastating emotional burden that infertility can cause on those who must live with it after surviving a cancer. It is as if the continuity of life itself be returned to the person, but without the perpetuation of one's memory or legacy through descendants.

In the surrounding context, oncofertility appears as an interdisciplinary field of study that combines expertise in reproductive medicine and oncology, to provide strategies aiming to maintain the possibility of future procreation (Woodruff, 2007). In practice, it involves a contingency plan, which aims to store gametes (oocytes and sperm) before they suffer potentially irreversible damage by chemotherapy and/or radiotherapy, and thus offer patients some hope of having an offspring that inherits their genetic material.

Thus, when we talk about fertility preservation after cancer, the greatest benefits are the psycho-emotional ones, as the impossibility of biological maternity/paternity is a great anguish of concern for the individual with the disease, and highlights the impotence felt by those facing its potential severity. The literature suggests that survivors who do not receive information about the possibility of becoming infertile after antineoplastic therapy present highly significant emotional stress levels (Quinn *et al*., 2015; Levine *et al*., 2015).

Considering the vulnerability and the immense psycho-emotional pressure resulting from the diagnosis of cancer, and its significant interference in the understanding and acceptance of patients and their families, interdisciplinarity is fundamental to the proper approach in oncofertility. Psychologists and social assistants, as well as family and friends, are the pillars without which no strategy can be sustained. Reproductive medicine opens the way for it, but the starting point is always in the hands of oncologists. So, it is time to involve them in discussing issues related to fertility preservation of their patients as part of the first line of approach, soon after diagnosis (Carvalho, 2015).

The best way to raise oncologists' awareness about the importance of oncofertility is to find out how many individuals would potentially benefit from fertility preservation strategies. Considering the estimate that 10% of female cancer cases occur before the age 45, with a survival rate of about 85% (Howlander *et al*., 2012.), and the annual statistics for cancer (Ferlay *et al*., 2010; Howlander *et al*., 2012; Ferlay *et al*., 2013; Ministry of Health - Brazil, 2014), one could deduce that more than 15 thousand women in Brazil, more than 66,000 in the US, more than 160,000 in Europe, and more than 830,000 women worldwide could benefit from being informed annually.

## FERTILITY PRESERVATION TODAY

Today we have many options and techniques available for the preservation of gametes for men and women. Some of them are already considered well established and used in routine, such as cryopreservation of semen, oocytes and embryos.

Semen cryopreservation is the classically used technique for preserving fertility in males. Semen samples are usually collected by masturbation, but surgically obtained sperm is accepted as a strategy in azoospermic individuals. Considering the sum of all the techniques, success rates vary around 40% (Bizet *et al*., 2012; Osterberg *et al*., 2014).

Cryopreservation of mature oocytes is the technique of choice in women with cancer (Loren *et al*., 2013). Fortunately, today we can already say that pregnancy rates obtained from frozen eggs are very similar to those obtained from fresh eggs, as well as the health of children conceived from the fertilization of frozen-thawed oocytes is similar. According to recent data, *in vitro* fertilization of frozen-thawed oocytes results in implantation and pregnancy from 10% to 60%, and from 30% to 60%, respectively, which are very close to rates obtained with fresh eggs (Cobo *et al*., 2013)

This brings safety to reproductive medical services and eliminates ethical conflicts which should be considered for the cryostorage of embryos from patients with a real risk of death. It happens that, to provide potentially better outcomes, ovulation induction is mandatory, and with it, the risks related to postponing the beginning of the antineoplastic treatment are raised, as well as the adverse effects of ovarian stimulation in favoring growth of estrogen-dependent tumors.

Although uncertainties are still common among oncologists, we can happily say that today they are not endorsed anymore, since the advent of random-start treatment protocols, not only allowing the patient to continue cancer treatment in two to three weeks after the first query (Cakmak *et al*., 2013a), but also being carried out in association with aromatase inhibitors (Cakmak & Rosen, 2013b) or selective estrogen receptors modulators (Franco *et al*., 2012), which reduces or eliminates the risk of hormonal-dependent tumor stimulation. Current literature provides promising results with random-start protocols and, although studies are still small and with low levels of evidence, many consider the data strong enough to encourage oncologists refer their patients to an oncofertility specialist early on (Cakmak *et al*., 2013a; Rashidi *et al*., 2014; Checa *et al*., 2015; Kim *et al*., 2015).

Ovarian cortex cryopreservation also takes its place among fertility preservation strategies, especially in specific situations, such as cancer in children. Despite being considered an experimental approach, sixty births have been well documented in the literature (Donnez & Dolmans, 2015), obtained after the orthotopic transplantation of cryopreserved tissue or transplant to places distant from the ovarian fossa. This technique is expected to soon rise from the experimental level to the routine of specialized services, although several ethical and moral issues need to be debated on the subject.

Finally, ovarian stimulation in women that have already undergone antineoplastic therapy results in reduced numbers of retrieved eggs. Additionally, there is a potential deleterious effect on the quality of those eggs and the embryos generated from them. Thus, the literature does not support cryopreservation of gametes in women who have already received anticancer treatment, especially chemotherapy, and such practice is not recommended (Koch & Ledger, 2013).

## ETHICAL AND MORAL ISSUES PERMEATING ONCOFERTILITY

Gamete or gonads storage for long periods generates ethical and moral questions without answers so far, but they deserve attention, reflection and discussion before one opts for a fertility preservation protocol. One reason for such discussion is the existence of uncertainties curtailing the processes that involves routine and experimental strategies, as well as the future use of the preserved tissues and cells in the face of the possibility of the death of their biological owner.

For how long cryopreserved biological material will be feasible? Although cryopreserved sperm can remain viable for many years (Bizet *et al*., 2012) and ovarian tissue may resist freezing, at least in the short term (Campos *et al*., 2011), experience with these and other techniques is too recent to ensure its security and its routine use. Will the freezing and thawing processes affect the quality and function of cells and tissues? Is it safe to use them? These are questions that only time will answer.

While recognizing the merits of the cause, questions arise from the indication of fertility preservation protocols for individuals with cancer. This is so because, from a technical point of view, those are treatments primarily linked to the diagnosis of infertility, and cancer patients are not necessarily infertile at the time of treatment or will be infertile after its completion, challenging the existence of a true medical indication (Basco *et al*., 2010). On the other hand, the provision of strategies to preserve fertility in the presence of any disease or treatment that may affect it reaches a moral obligation level, respecting autonomy of choice, which is an essential foundation of a free society (Larcher, 2012).

Thus, it is understood that fertility preservation preceding antineoplastic treatment lays between medical indication - based on the intention of prevention -, humanization, and a social statement - based on biopsychosocial impact of procreating disability. In the case of cancer patients with potential risk of fertility loss, the real ethics is to furnish the best information about the potential risks and the currently available techniques for the preservation of their gametes. This will allow well-informed patients and their families to make the right decisions with the necessary clarity, based on personal interest concerning the possibility of future fertility.

Disquieting questions need to be raised to a profound and sensible approach to fertility preservation of anyone. In a very real sense for feminist authors, the observation that some medical procedures are considered as solutions to social problems is seen often with pejorative eyes. One of the questions is related to the possible inappropriate social pressure on women with cancer or parents of girls and adolescents with cancer, because of the obligation of future motherhood as a requirement of society (Traina, 2010).

Finally, the universal right to procreate gives ethical, legal and moral support for the development of oncofertility. Similarly, the principles of autonomy, beneficence and non-maleficence should chart the course of the chosen strategy with respect and attention, including children and adolescents' opinion, when they can understand the circumstances (Carvalho, 2015).

## FINAL CONSIDERATIONS

It is very likely that most clinics offering fertility preservation strategies to individuals with cancer have little experience with successful pregnancies, as we still know very little about the long-term viability of their cryopreserved oocytes and ovarian or testicular tissues. The long-term risks of the procedures related to fertility preservation are also not well known.

However, there is no doubt that when we talk about fertility preservation after cancer, there are great psycho-emotional gains involved, since the feeling of discontinuity in not being able to leave a biological legacy is a source of great distress, and highlights personal impotence in facing a life-threatening disease. Thus, without the intention to offer a guarantee, but a chance of procreation to cancer patients in a possible disease-free future, oncofertility is a reality and there is no reason supporting the lack of early information on it in a moment after cancer confirmation.

It is expected that soon oncofertility will play its role as a modifier of medical culture, innovating in that it opens the eyes of the world to the expectation of procreation as a quality factor of survival to cancer.
